# Thermally Activated vs. Photochemical Hydrogen Evolution Reactions–A Tale of Three Metals

**DOI:** 10.1002/chem.202203590

**Published:** 2023-03-22

**Authors:** Milan Ončák, Chi‐Kit Siu, Christian van der Linde, Wai Kit Tang, Martin K. Beyer

**Affiliations:** ^1^ Universität Innsbruck Institut für Ionenphysik und Angewandte Physik Technikerstraße 25 6020 Innsbruck Austria; ^2^ Department of Chemistry City University of Hong Kong 83 Tat Chee Avenue, Kowloon Tong Hong Kong SAR P. R. China; ^3^ Institute of Research Management and Services (IPPP) Research and Innovation Management Complex University of Malaya Kuala Lumpur 50603 Malaysia

**Keywords:** hydrated metal ions, hydrogen evolution, mass spectrometry, photochemistry, spectroscopy

## Abstract

Molecular processes behind hydrogen evolution reactions can be quite complex. In macroscopic electrochemical cells, it is extremely difficult to elucidate and understand their mechanism. Gas phase models, consisting of a metal ion and a small number of water molecules, provide unique opportunities to understand the reaction pathways in great detail. Hydrogen evolution in clusters consisting of a singly charged metal ion and one to on the order of 50 water molecules has been studied extensively for magnesium, aluminum and vanadium. Such clusters with around 10–20 water molecules are known to eliminate atomic or molecular hydrogen upon mild activation by room temperature black‐body radiation. Irradiation with ultraviolet light, by contrast, enables hydrogen evolution already with a single water molecule. Here, we analyze and compare the reaction mechanisms for hydrogen evolution on the ground state as well as excited state potential energy surfaces. Five distinct mechanisms for evolution of atomic or molecular hydrogen are identified and characterized.

## Graphical abstract

**All roads lead to hydrogen**. an in‐depth analysis and comparison of hydrogen evolution reactions in gas phase model systems reveal five distinct mechanistic routes to form either atomic or molecular hydrogen.

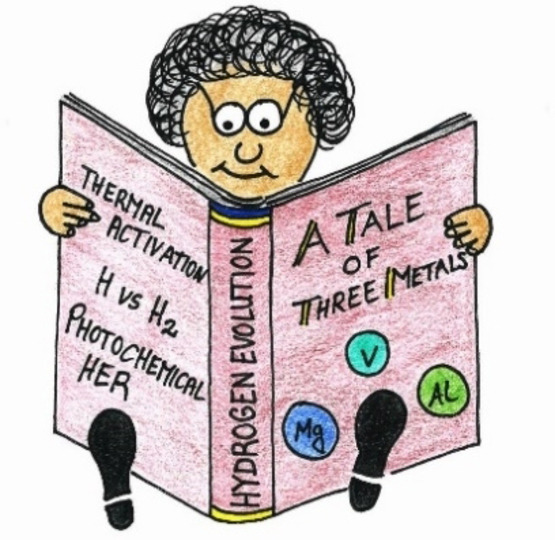

## 1 Introduction

Hydrogen plays an important role in concepts for transforming the energy, transportation and chemical sectors towards a net‐zero‐CO_2_ economy.[^1^, ^2^] The hydrogen evolution reaction (HER) in electrochemical environments[^3^, ^4^, ^5^] as well as gas‐phase model systems[^6^, ^7^] are frequently studied to understand its mechanism and improve the efficiency of hydrogen generation for purposes like energy storage,^[8]^ transportation[^9^, ^10^] or decarbonization of large‐scale CO_2_ releasing processes, for example steel production^[11]^ or ammonia synthesis.^[12]^

Starting with the discovery of water electrolysis by Carlisle and Nicholson in 1800,^[13]^ hydrogen evolution reactions have also sparked the curiosity of researchers. The high‐school experiment, in which hydrogen is generated when a piece of alkaline metal reacts with water, received renewed attention through the experiment by Jungwirth and colleagues.^[14]^ They showed that the fast release of solvated electrons leads to a Coulomb explosion of the metal, which precedes hydrogen formation. In the gas phase, formation of a solvated electron was observed in water clusters with an excess sodium atom by Schulz et al.,^[15]^ starting with as few as four water molecules. Molecular hydrogen evolution was observed by Buck and co‐workers in sodium‐water clusters with three or five sodium atoms,^[16]^ with theoretical explanations contributed by Parrinello and coworkers.^[17]^

Gas‐phase hydrated metal ions in unusual oxidation states are particularly useful model systems for detailed investigation of hydrogen evolution reactions, since they can be studied under idealized conditions in ultra‐high vacuum by mass spectrometric methods.^[18]^ They can be subjected to collision induced dissociation with controlled kinetic energy[^19^, ^20^] or gently heated by black‐body infrared radiative dissociation (BIRD) while trapped in a Fourier‐Transform Ion Cyclotron Resonance (FT‐ICR) mass spectrometer.[^21^, ^22^, ^23^, ^24^] Combined with laser photodissociation spectroscopy in the ultraviolet (UV), visible (Vis) and infrared (IR) region and quantum chemical calculations,[^25^, ^26^] a wealth of information on the structure, energetics and reaction mechanisms can be obtained.

To be able to compare hydrogen evolution in the ground state with photochemical excited state reactions, we focus here on hydrated metal ions which are known to undergo thermally activated hydrogen evolution reactions. Other metals, however, may very well exhibit photochemical hydrogen evolution, as known, for example, for [Fe(H_2_O)_*n*_]^+^.[^27^, ^28^] Further complexity can be expected upon introduction of several metal centers into a water cluster, for which the recently studied vanadium cluster‐water reactions are one example,[^29^, ^30^] or by adding C_60_ to a V^+^(H_2_O) complex.^[31]^ Uptake of reactants may also trigger hydrogen evolution in otherwise unreactive systems, as observed for [Zn(H_2_O)_*n*_]^+^ in collisions with HCl.^[32]^ Water activation may proceed without subsequent hydrogen evolution, as documented for [Mn(H_2_O)_*n*_]^+^.[^33^, ^34^]

This reductionist approach of course comes at a price. The reaction mechanisms obviously cannot be directly transferred to electrochemical environments, where controlled electric potentials are applied, and interface effects at extended surfaces play an important role. In the gas phase clusters discussed here, the metal ion is in a lower than preferred oxidation state, and thus is able to provide one or two electrons for redox chemistry. The mechanistic insight that can be gained from gas phase studies, however, may inspire practitioners in their quest of developing more efficient catalysts and processes both for the production of hydrogen in electrolyzers and its conversion back to water in fuel cells.

For this concept article, we single out three extensively investigated hydrated metal centers, namely [Mg(H_2_O)_*n*_]^+^, [Al(H_2_O)_*n*_]^+^ and [V(H_2_O)_*n*_]^+^, to compare their ground‐state reactivity with excited‐state photochemistry. The metal ions were chosen due to their different electronic structure that enables us to explore hydrogen evolution under various conditions. The Mg^+^ ion with its 3 s^1^ configuration is isoelectronic to a sodium atom and one can expect its chemistry to be driven by the unpaired, loosely bound electron. The Al^+^ ion has a closed 3 s sub‐shell, and should be thus considerably less reactive compared to Mg^+^. Finally, V^+^ with its electronic configuration of [Ar] 3d^4^ represents a prototypical transition metal ion. It is the only first row transition metal ion M for which we obtained clusters of the composition [M(H_2_O)_*n*_]^+^, and which at the same time exhibits hydrogen formation via BIRD at room temperature. These systems exhibit an unexpectedly rich chemistry, illustrating the diverse mechanisms that can be operative in hydrogen evolution reactions.

## 2 Experimental and Theoretical Methods

All experimental results were obtained on a Bruker/Spectrospin CMS47X FT‐ICR mass spectrometer, equipped with a laser vaporization source.^[35]^ Hydrated ions are produced by laser vaporization of a solid disk of the respective metal, followed by supersonic expansion of the hot plasma in a short pulse of the helium carrier gas, which is seeded with traces of water. Cluster ions are transferred from the source chamber by a set of electrostatic lenses via several differential pumping stages to the ICR cell, where they can be stored for an arbitrary period of time. A typical BIRD experiment is performed by recording mass spectra after variable storage times at room temperature.

To perform photodissociation spectroscopy, the ICR cell is cooled with liquid nitrogen to about 80–100 K, which largely suppresses BIRD.^[24]^ Light from a tunable laser system, featuring an optical parametric oscillator and frequency doubling as well as sum frequency mixing stages to cover the wavelength range of 225–2600 nm (EKSPLA NT342B), was introduced into the ICR cell via a mechanical shutter. Cluster ions are mass selected, irradiated with a defined number of laser pulses, and a mass spectrum of the remaining original ions and the photodissociation products is recorded. This procedure is repeated for a series of wavelengths to obtain the data points for a photodissociation spectrum. Details on data analysis and derivation of photodissociation cross sections are found in the original publications.[^36^, ^37^, ^38^]

Accurate computational approaches are indispensable for the description of the electronic structure and reactivity of the clusters. For ground state and IR calculations, density functional theory (DFT) and coupled cluster (CC) approaches are among the most popular ones. For excited states, time‐dependent DFT (TD‐DFT) and equation of motion CC (EOM‐CC) are often used. For transition metals and molecules with complex electronic structure, multi‐reference approaches have to be employed, based on the complete active space self‐consistent field scheme (CASSCF), often followed by application of perturbation treatment or multi‐reference configuration interaction (MRCI) calculation. The choice of the specific method and basis set used for each of the studied systems was guided by extensive benchmarking and computational cost. For example, small clusters with the main group elements magnesium and aluminum can be treated at a higher computational level than large clusters containing a vanadium ion with its open 3d shell.

## 3 Magnesium

### 3.1 Ground‐state reactivity

**Figure 1:**
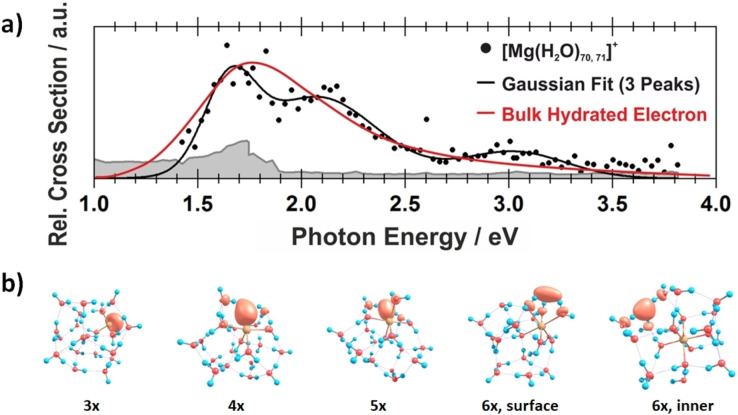
a) Photodissociation spectrum of [Mg(H_2_O)_*n*_]^+^, *n*=70, 71, compared with the absorption spectrum of the bulk hydrated electron.^[45]^ b) Calculated structures of *n*=20 with increasing coordination number (CAM‐B3LYP/aug‐cc‐pVDZ//ωB97XD/def2TZVP). Adapted under CC‐BY license from Ref. [44]. © 2019 The Authors.

Mass spectra of hydrated magnesium ions show three regions, separated by smooth boundaries.[^39^, ^40^, ^41^, ^42^, ^43^] For *n*≤5, [Mg(H_2_O)_*n*_]^+^ are observed, which are genuinely monovalent magnesium ions solvated with water molecules in the first or second solvation shell. The intermediate size regime, 6≤*n*≤16, is dominated by [MgOH(H_2_O)_*n‐*1_]^+^. For *n*≥17, again [Mg(H_2_O)_*n*_]^+^ ions are identified in the mass spectrum. This time, however, we are dealing with a Mg^2+^ ion and a hydrated electron, which may form a contact ion pair Mg^2+^e^−^(H_2_O)_*n*_ or solvent‐separated ion pair Mg^2+^(H_2_O)_*n*_e^−^, with the electron staying in the vicinity of the doubly charged metal center.^[44]^ Figure 1 shows an experimental electronic absorption spectrum, which is close to the spectrum of the bulk hydrated electron, together with quantum chemical calculations that illustrate the diffuse molecular orbital of the unpaired electron in the cluster. The absorption features are tentatively assigned to at least two structural isomers, which differ either in the position of the hydrated electron or in the coordination number of the magnesium center, or both.

It is quite intriguing that this structural feature of the large clusters was inferred correctly by Fuke and Iwata, based alone on the time of flight mass spectra.^[46]^ Niedner‐Schatteburg et al. presented an elaborate thermochemical cycle to show that formation of a hydrated Mg^2+^/e^−^ pair is energetically favorable in bulk aqueous solution.^[41]^ Reinhard and Niedner‐Schatteburg^[47]^ as well as Siu and Liu[^48^, ^49^] later conducted quantum chemical calculations that corroborated these ideas, showing the spin density of the hydrated electron is displaced from the magnesium center for *n*≥6. The presence of the hydrated electron in large clusters is also evidenced by the reactivity of [Mg(H_2_O)_*n*_]^+^, which exhibit the chemistry of the hydrated electron in reactions with CO_2_, O_2_, and CH_3_CN.[^50^, ^51^, ^52^, ^53^, ^54^, ^55^, ^56^, ^57^, ^58^]

Fuke, Iwata and co‐workers interpreted their time of flight mass spectra in terms of a reaction of Mg^+^ ions with neutral water clusters (H_2_O)_*n*_.^[46]^ They showed by quantum chemical calculations that [MgOH(H_2_O)_*n*‐1_]^+^+H is thermochemically favored over [Mg(H_2_O)_*n*_]^+^ for *n*≥5. Niedner‐Schatteburg, Bondybey and co‐workers showed that mass‐selected [Mg(H_2_O)_*n*_]^+^, *n*≈16–21, eliminate H atoms together with a water molecule under the influence of room temperature black‐body radiation, reaction (1).[^41^, ^43^] The mechanism of this reaction is a recombination of the hydrated electron with a proton, which is formed by the strongly polarizing effect of the small Mg^2+^ ion,[^41^, ^43^, ^46^] transiently involving a salt‐bridge arrangement Mg^2+^(OH^−^)(H_3_O^+^) which originates from water molecules in the first and second solvation shell.[^43^, ^49^, ^59^, ^60^] When the cluster size increases, the reaction becomes less thermochemically favorable, and the hydrated electron moves further away from the metal center,^[49]^ which leads to the gradual switching‐off of the reaction for *n*≥22., ^[43]^

(1)

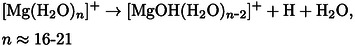




This interpretation gains additional support by reactivity experiments with HCl. Upon uptake of an HCl molecule by gas phase hydrated electrons (H_2_O)_*n*_^−^, the molecule dissociates ionically. The proton recombines with the electron, and an H atom leaves the cluster, with the Cl^−^(H_2_O)_*m*_ product observed in the mass spectrum.^[61]^ The analogous chemistry takes place with [Mg(H_2_O)_*n*_]^+^, *n*≥17, with formation of [MgCl(H_2_O)_*m*_]^+^ and release of a hydrogen atom.^[43]^

### 3.2 Photochemistry

Large [Mg(H_2_O)_*n*_]^+^ clusters, which contain Mg^2+^ and a hydrated electron, respond to photoexcitation by water evaporation.^[44]^ This closely mirrors the behavior of gas‐phase hydrated electrons, which undergo ultrafast internal conversion on a sub‐ps time scale.[^62^, ^63^] The entire photon energy is converted to heat, which causes evaporation of several water molecules. No photochemical hydrogen evolution is observed for [Mg(H_2_O)_*n*_]^+^ in the size regime *n*≥17. In the intermediate size regime with *n* ≈6‐16, where only hydroxide species [MgOH(H_2_O)_*n*‐1_]^+^ are observed, calculated electronic excitations lie outside the accessible wavelength range (<200 nm).

The structure of the smallest clusters with *n*≤5 has been characterized by infrared spectroscopy[^64^, ^65^] and quantum chemistry.[^66^, ^67^, ^68^] In the electronic ground state, no hydrogen evolution is observed. Collision induced dissociation (CID) proceeds exclusively via loss of intact water molecules, as reported by Armentrout and co‐workers.[^69^, ^70^] By contrast, all clusters with *n*=1‐5 exhibit H atom loss upon electronic excitation.[^38^, ^39^, ^40^, ^71^] This comparison immediately suggests that H atom loss occurs exclusively in an electronically excited state. The dominant electronic excitation was assigned to 3s–3p transitions of the Mg^+^ center,[^39^, ^40^, ^71^] confirmed with multi‐reference configuration interaction (MRCI) calculations by Watanabe and Iwata.^[72]^

**Figure 2:**
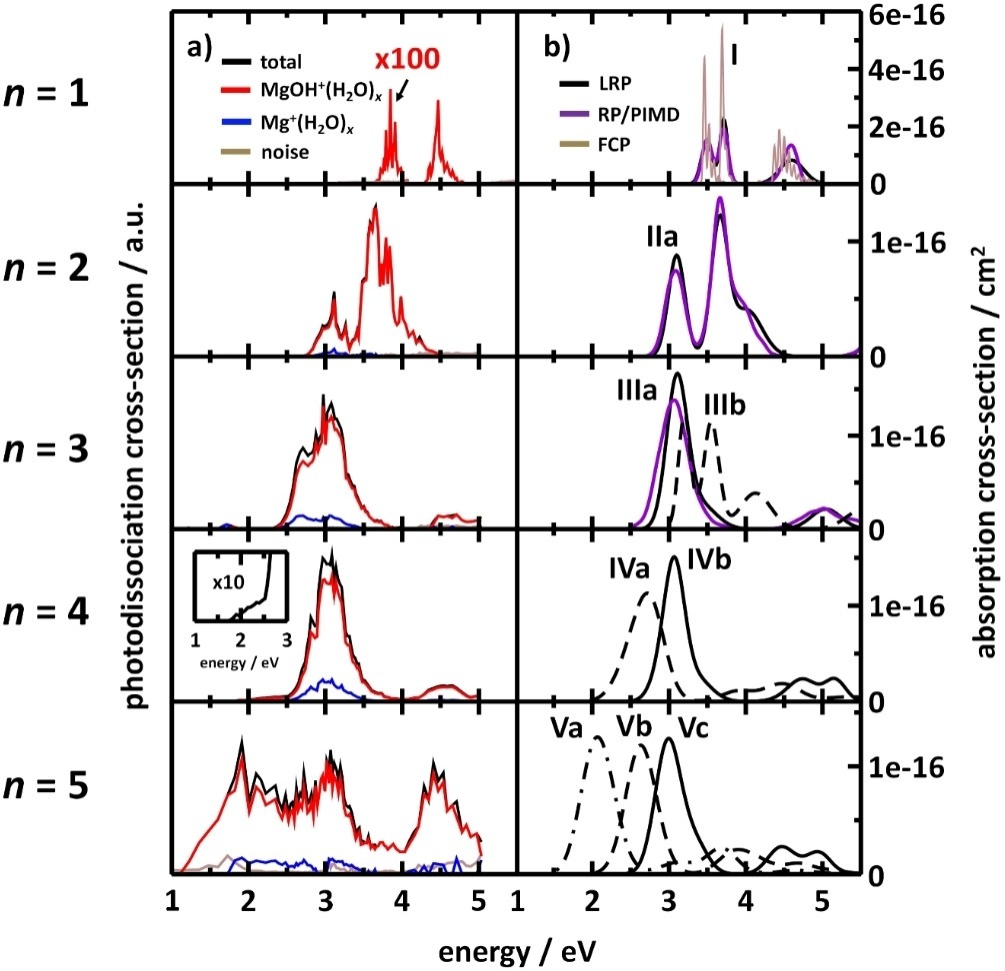
Photodissociation and photoabsorption spectra of [Mg(H_2_O)_*n*_]^+^, *n*=1–5, clusters. a) Experimental photodissociation spectra. For *n*=4, the spectral onset is also shown, with 10x higher intensity. b) Modeled photoabsorption spectra using linearized reflection principle (LRP; CC2/aug‐cc‐pVTZ//(TD−)CAM−B3LYP/aug‐cc‐pVTZ), reflection principle with path integral molecular dynamics (RP/PIMD; CC2/aug‐cc‐pVDZ//B3LYP/6‐31+g*), and Franck‐Condon principle (FCP; EOM‐CCSD/aug‐cc‐pVDZ). Clusters are denoted according to decreasing coordination of Mg^+^; for example, **Va** and **Vb** include five and four water molecules in the first solvation shell, respectively. Adapted under CC‐BY license from Ref. [38]. © 2018 The Authors.

Our recent photodissociation spectra of [Mg(H_2_O)_*n*_]^+^, *n*=1‐5, are shown in Figure 2, together with simulated spectra based on high‐level quantum chemical calculations. Loss of atomic hydrogen is the dominant photodissociation channel for all sizes. The nearly complete absence of H_2_O loss indicates that internal conversion to the electronic ground state is inefficient or even impossible.

The first band in the *n*=1 spectrum is very weak, because two photons are needed to excite the system above the dissociation threshold. Multiple bands are observed because the degeneracy of the ^2^P atomic term is lifted by the interaction with the water molecule. Each additional water molecule in the first solvation shell causes a significant redshift. Starting with *n*=3, 3s–3d/4 s transitions emerge above 4 eV. For *n*=5, a mixture of three‐, four‐ and five‐fold coordination is present in the experimental cluster population. This is consistent with quantum chemical calculations, which indicate that the lowest lying isomers featuring these coordination numbers lie within 4 kJ mol^−1^.^[38]^ Our spectra calculated with the linearized reflection principle (LRP) on the CC2/aug‐cc‐pVTZ level of theory reproduce the experimental spectra very well, both in terms of absolute band position and intensities.

**Figure 3:**
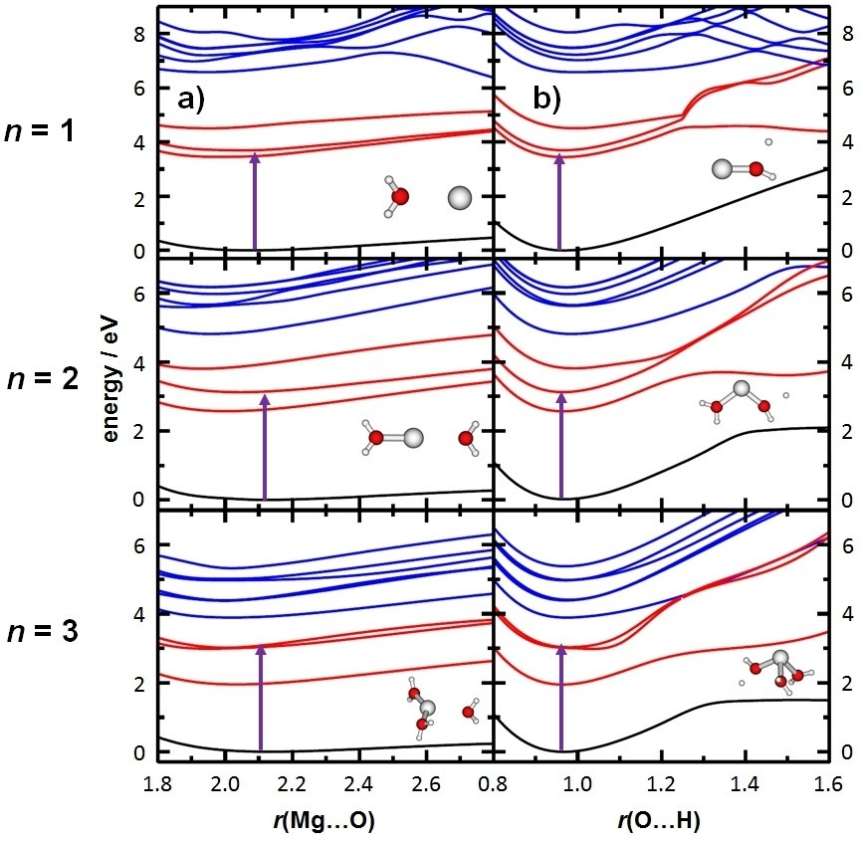
Relaxed potential energy scans for [Mg(H_2_O)_*n*_]^+^, *n*=1–3, for a) Mg−O and b) O−H dissociation coordinates (EOM‐CCSD/aug‐cc‐pVDZ). The clusters were optimized in the D_0_ (black lines), D_1_ (red lines) and D_4_ (blue lines) electronic states. Pre‐dissociated ions are shown for calculations in the D_1_ state. The D_0_‐D_1_ excitation energy (violet arrow) was calculated in the structure optimized at the MP2/def2TZVP level. Adapted under CC‐BY license from Ref. [38]. © 2018 The Authors.

Possible photochemical reaction pathways are analyzed by relaxed scans along two reaction coordinates representing the observed dissociation pathways, Mg−O and O−H, as illustrated by selected structures along the reaction paths in Figure 3. In all cases, there is a significant gap between the ground state, D_0_, and the first excited state, D_1_, where D refers to the doublet spin multiplicity of the studied species. This indicates that internal conversion to the ground state is not possible, since the required conical intersection is not there, which explains the near absence of water loss as a photodissociation channel. Red and blue lines refer to excited 3p and 3d/4 s states of the Mg^+^ center, respectively. Overall, it seems plausible that conical intersections for radiationless relaxation between excited state potential energy surfaces (PESs) are more readily reached with elongated O−H bonds. In favorable cases, repulsive sections of the PES can be reached that afford H atom loss, while all but one potential curves are attractive along the Mg−O coordinate. With the O−H bond elongating, the spin density localizes on the H atom, and the metal center is oxidized to Mg^2+^, representing electron transfer from Mg^+^ to a water molecule.

Obviously, this photochemical reaction pathway for *n*≤5 is fundamentally different from the ground state reactivity; (i) no hydrated electron is required and (ii) the reaction is endothermic. For *n*=1, close to 3 eV of the photon energy are retained as chemical energy in thermalized reaction products, but this value quickly decreases to 0.24 eV at *n*=5.^[38]^ Although loss of water is by far the energetically preferred pathway for cluster sizes *n*=1‐3, H atom loss is predominantly observed.

## 4 Aluminum

### 4.1 Ground‐state reactivity

In contrast to [Mg(H_2_O)_*n*_]^+^, hydrated aluminum ions [Al(H_2_O)_*n*_]^+^ react by elimination of molecular hydrogen. The reaction proceeds on a timescale of seconds under the influence of room‐temperature black‐body radiation and can be monitored by FT‐ICR mass spectrometry.[^73^, ^74^] It exhibits a very peculiar size dependence, occurring only for *n*=11‐24, with a pronounced maximum in the rate and branching ratio for *n*=20, 21.^[74]^

Extensive quantum chemical studies by Reinhard and Niedner‐Schatteburg^[75]^ as well as Siu, Liu and Tse^[76]^ revealed that the reaction proceeds in two distinct steps. In the rearrangement to [HAlOH(H_2_O)_*n*‐1_]^+^, reaction (2), the aluminum center is oxidized from +I to its preferred +III oxidation state. Subsequent H_2_ elimination, reaction (3), is a comproportionation of H^−^ and H^+^ to form H_2_, which can be viewed as an acid‐base reaction,^[75]^ but also as a redox reaction.
(2)

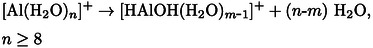



(3)

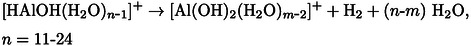




For *n*=20, the inserted [HAlOH(H_2_O)_*n*‐1_]^+^ isomer was calculated by Reinhard and Niedner‐Schatteburg to be 194 kJ mol^−1^ lower in energy, and the barrier for reaction (2) is with 14 kJ mol^−1^ relatively small. Elimination of molecular hydrogen from a slightly higher‐lying [HAlOH(H_2_O)_*n*‐1_]^+^ isomer is −122 kJ mol^−1^ exothermic, with a barrier of 45 kJ mol^−1^.^[75]^ Given the huge number of possible isomers, there may be other isomers yielding slightly different numbers, but the overall energetics of the two reactions is realistic. Siu, Liu and Tse conducted extensive ab initio molecular dynamics (AIMD) simulations on a series of smaller clusters, which provided additional insight.^[76]^ These simulations predicted that the structure of the [HAlOH(H_2_O)_*n*‐1_]^+^ cluster starts with the hydride on the cluster surface at *n*=6. Upon increasing solvation, a caged structure gradually evolves, with the Al−H bond connected to other water molecules via hydrogen bonds, to be completed at *n*=13.

**Figure 4:**
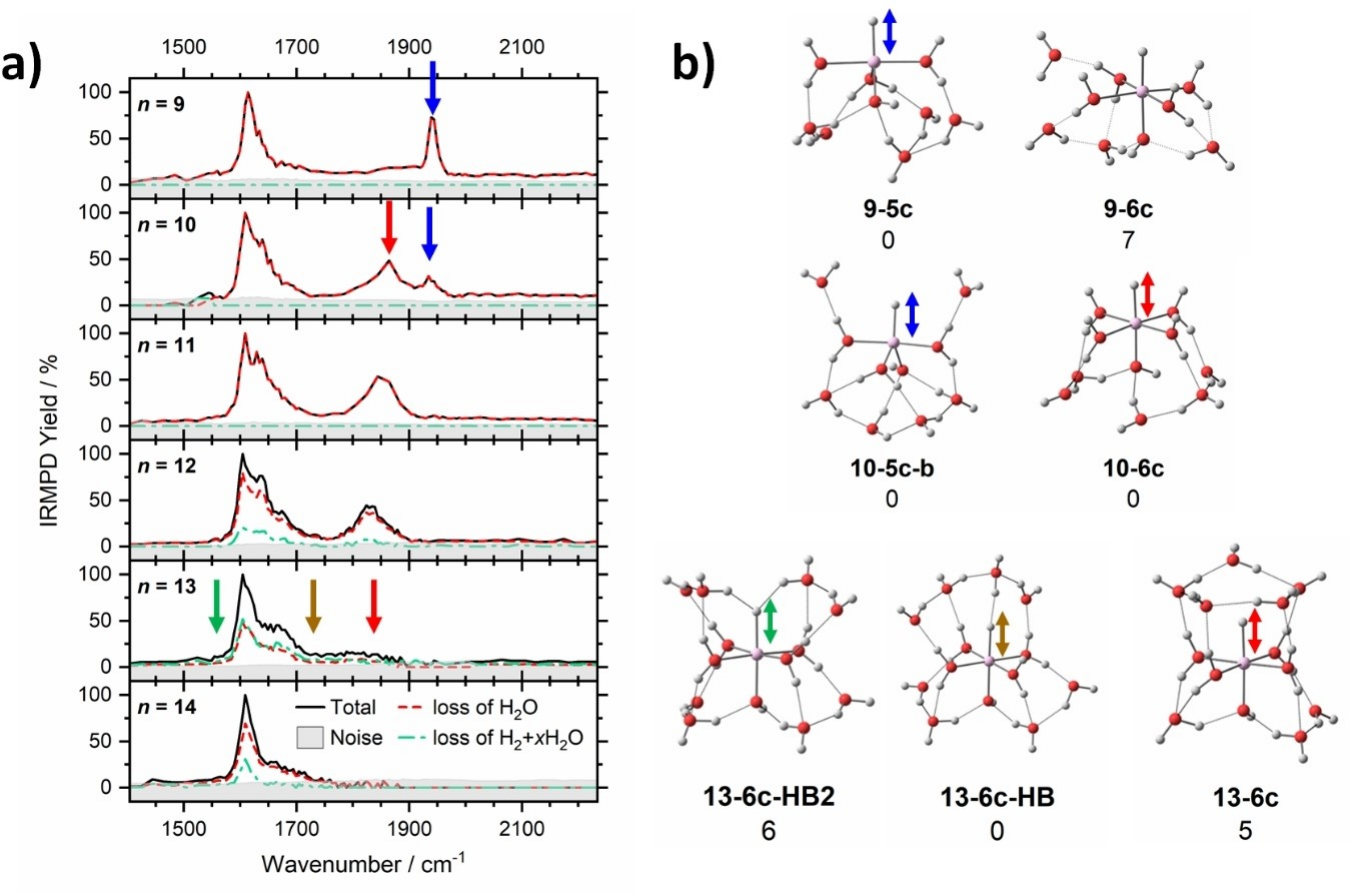
a) Experimental IRMPD spectra of [Al (H_2_O)_*n*_]^+^, *n*=9–14. b) Low‐lying isomers of [HAlOH(H_2_O)_*n*‐1_]^+^ with five‐ and six‐fold coordinated metal center (X‐5c, X‐6c) and one or two hydrogen bonds (HB, HB2) to the hydride as calculated at the M06/6‐311++G** level. Positions of the Al−H stretch are marked by arrows. Adapted under CC‐BY license from Ref. [77]. © 2021 The Authors.

We recently confirmed this prediction by infrared multiple photon dissociation (IRMPD) spectroscopy.^[77]^ Figure 4 shows IRMPD spectra of [HAlOH(H_2_O)_*n*‐1_]^+^, *n*=9‐14, in the Al−H stretch/H_2_O bend region. It is clearly visible that the Al−H stretch frequency shifts to the red with increasing coordination number. Hydrogen bonding causes additional redshift and, due to the fluxional nature of the hydrogen bonded water network, substantial broadening. The hydride may even act as a double acceptor, causing the Al−H stretch frequency to shift below the water bending mode.

The reaction mechanism of hydrogen evolution in the [Al(H_2_O)_*n*_]^+^ system is thus firmly established. First, a concerted proton transfer through a water wire takes place, and the proton attacking the metal center accepts the electron pair from the Al 3 s orbital to form the hydride in the inserted [HAlOH(H_2_O)_*n*‐1_]^+^ species. In the second step, proton/hydride recombination via a second concerted proton transfer leads to the evolution of molecular hydrogen and formation of hydrated dihydroxide [Al(OH)_2_(H_2_O)_*n*‐2_]^+^. The reason for the absence of H_2_ elimination beyond *n*=24, however, is less well understood. The upper limit is shifted to larger clusters by reacting [Al(H_2_O)_*n*_]^+^/[HAlOH(H_2_O)_*n*‐1_]^+^ species with methanol,^[78]^ and all studied clusters eliminated H_2_ in reaction with formic acid or HCl.[^74^, ^78^] Siu and Liu argued that the probability for H^+^/H^−^ recombination decreases dramatically in larger clusters.^[76]^ Our D_2_O exchange experiments[^79^, ^80^] with large [Al(H_2_O)_*n*_]^+^/[HAlOH(H_2_O)_*n*‐1_]^+^ showed that proton transfer does not take place in clusters with *n*>38, but the connection of this observation to the upper limit of H_2_ evolution with *n*≤24 remains elusive.

### 4.2 Photochemistry

Fuke and co‐workers reported photodissociation of [Al(H_2_O)_*n*_]^+^.^[81]^ At 248 nm, they observed strong fragments for *n*=2–6 and few weak features up to *n*=10. They identified water loss and no hydrogen evolution, but the peaks in the mass spectra look relatively broad. For *n*=2 and 3, a section of the photodissociation spectrum could be obtained, with an onset around 5.2 eV and 4.7 eV, respectively. In analogy to [Mg(H_2_O)_*n*_]^+^, the absorption was attributed to the 3s–3p transition of the Al^+^ metal center.

We recently revisited this system and were able to measure photodissociation spectra down to 225 nm (up to 5.51 eV) for [Al(H_2_O)_*n*_]^+^, *n*=1–10.^[37]^ For *n*=1, only one data point at 225 nm was obtained, for which we identified the photochemical loss of a hydrogen atom. For *n*=2–8, strong absorption is observed starting around 4.5 eV, with cross sections of the order of 10^−17^ cm^2^. Loss of atomic hydrogen, accompanied by water evaporation, is the dominant photodissociation pathway for *n*=2 and 3, while H_2_ formation and simple water elimination are observed in roughly equal amounts. With increasing cluster size, the branching ratio of H atom elimination decreases quickly, and simple water loss becomes the dominant photodissociation pathway, possibly accompanied by a conversion of [Al(H_2_O)_*n*_]^+^ to the thermochemically preferred inserted species [HAlOH(H_2_O)_*n*‐1_]^+^. Apparent absorption cross sections decrease with increasing cluster size, and the photodissociation signal completely disappears at *n*=9. Since the inserted species [HAlOH(H_2_O)_*n*‐1_]^+^ absorb only at much higher photon energies, this indicates that the non‐inserted species [Al(H_2_O)_*n*_]^+^ with *n*≥9 have a lifetime below 0.1 s at *T*∼83 K, the temperature of the ICR cell in the experiment. Again, CID studies by Armentrout and co‐workers report exclusively water loss for *n*=1–4,^[19]^ which shows that excited state chemistry is responsible for the observed light‐induced hydrogen evolution in this size regime.

**Figure 5:**
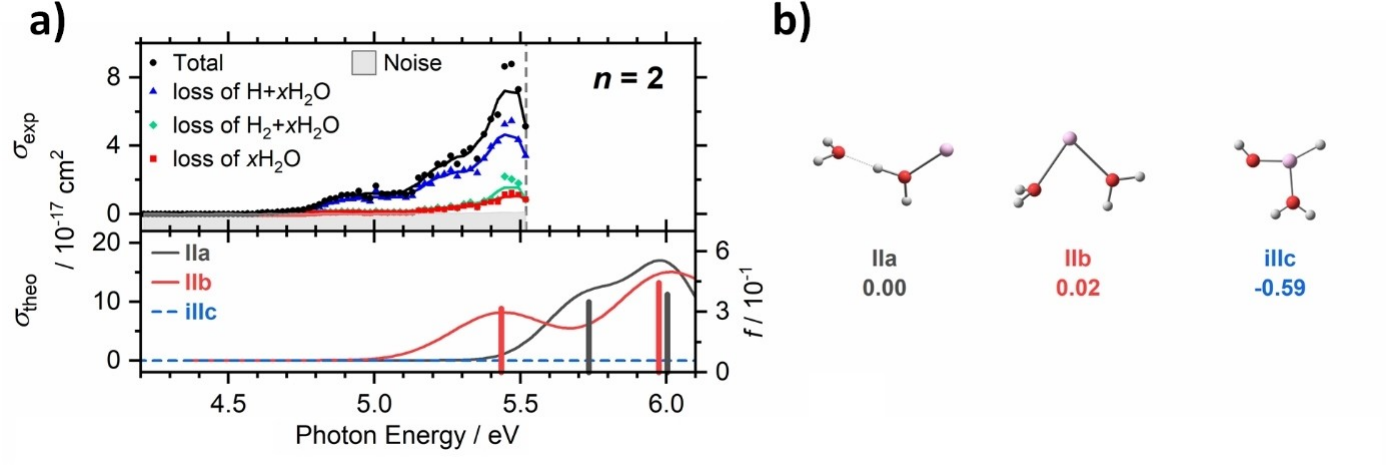
a) Upper panel: Measured photodissociation cross section for Al^+^(H_2_O)_2_ broken down into the chemically distinct channels. The border of the experimentally accessible energy range is marked by a dashed line. Lower panel: Modeled cross section and the corresponding oscillator strengths *f* (BHandHLYP/aug‐cc‐pVDZ//B3LYP/aug‐cc‐pVDZ), with spectral width estimated through linearized reflection principle. b) Located isomers along with relative energies in eV (B3LYP/aug‐cc‐pVDZ level). Adapted under CC‐BY license from Ref. [37]. © 2021 The Authors.

Figure 5 shows measured and simulated spectra for *n*=2. The simulation of the doubly coordinated **IIb** isomer is in excellent agreement with experiment, while the singly coordinated **IIa** isomer may contribute to a minor extent at the highest measured energies. Calculated absorptions of the inserted **iIIc** isomer lie well above the highest photon energies used in this work. As expected, the absorption is centered on the aluminum ion, and corresponds to a 3s–3p transition.

To get an idea about possible reaction mechanisms leading to H/H_2_ formation, we analyzed the reaction paths starting with insertion of the Al center into an O−H bond on the S_0_ and T_1_ potential energy surfaces. Several minima, denoted I1–I4, and their connecting transition states were localized, for details see Figure 3 in reference [37]. All the stationary points are consistently more stable in the singlet spin multiplicity. However, in the absence of a conical intersection that would connect S_1_ and S_0_, this approach failed to explain the observed photochemistry.

**Figure 6:**
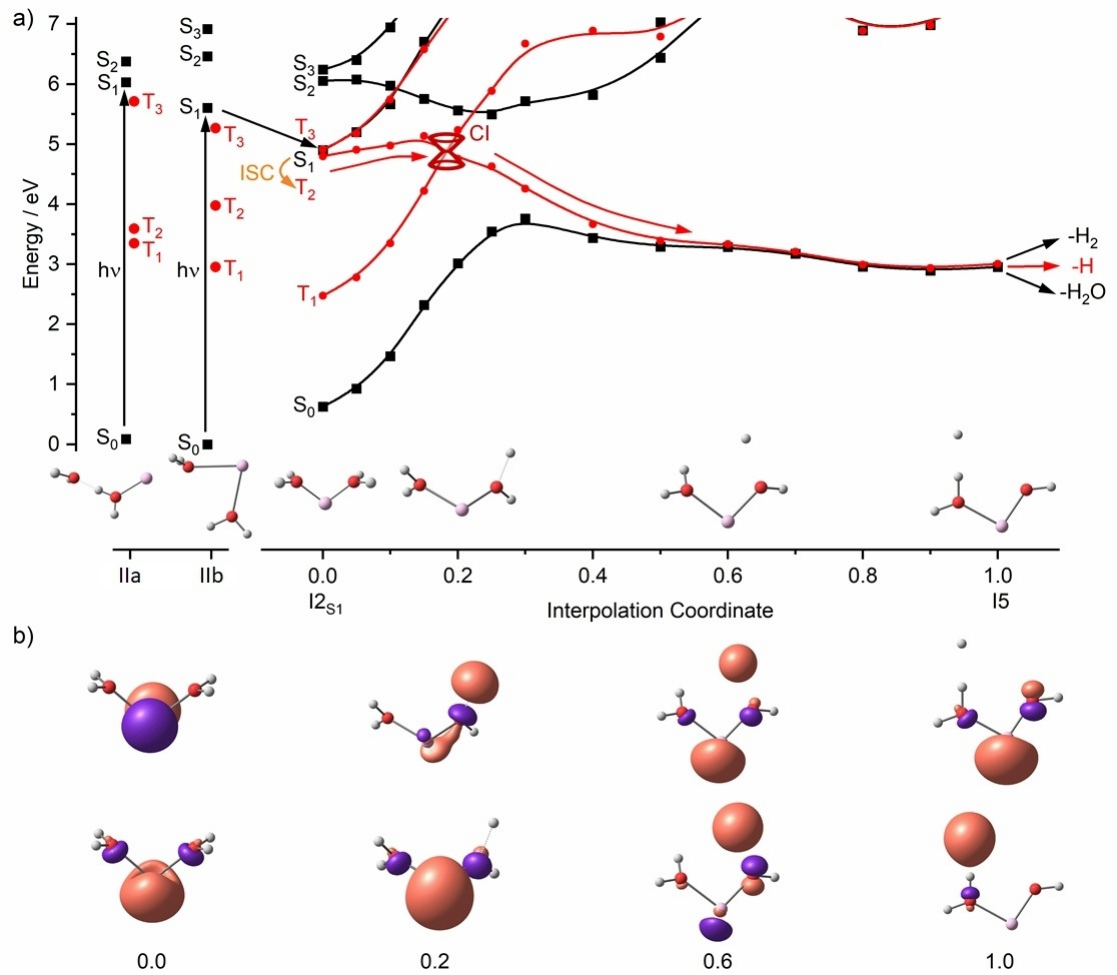
a) Calculated excitation energies in the Franck Condon point for Al^+^(H_2_O)_2_ in isomers **IIa** and **IIb**, relaxation into excited state minimum I2_S1_ of S_1_ (point 0.0) and interpolation to T_1_/S_0_ minimum I5 (point 1.0). I2_S1_ was optimized at the BHandHLYP/aug‐cc‐pVDZ level, other points at B3LYP/aug‐cc‐pVDZ level. Energies of electronic states were calculated at the MRCI(6,8)/aug‐cc‐pVDZ level without zero‐point correction. b) Natural transition orbitals for S_1_ at BHandHLYP/aug‐cc‐pVDZ (0.0) and the two highest‐lying alpha‐spin orbitals in T_1_ (0.2–1.0) at B3LYP/aug‐cc‐pVDZ at selected points along the interpolation coordinate. Reproduced under CC‐BY license from Ref. [37] © 2021 The Authors.

After an extensive search for local minima on various excited state potential energy surfaces, intermediate I5 was identified, which is a local minimum in the T_1_ state and has biradical character. Figure 6a shows an analysis of the rearrangements in the first excited state leading to I5, based on quantum chemical calculations. The absorbing species here is **IIb**, with both water molecules bound to Al^+^. Upon excitation, the molecule relaxes to I2_S1_, a local minimum on the S_1_ potential energy surface, which was localized by geometry optimization in the S_1_ excited state. Without change of the spin multiplicity, the system can relax only by fluorescence, since there is no easily accessible conical intersection connecting the S_1_ and S_0_ potential energy surfaces. The vibrational excitation remaining in the electronic ground state may lead to water evaporation. However, at I2_S1_, S_1_ and T_2_ states are almost isoenergetic, which makes intersystem crossing (ISC) feasible. The energy in the system is sufficient to surpass the small barrier towards the conical intersection (CI) with the T_1_ state along the I2_S1_‐I5 interpolation coordinate. Relaxation towards I5 is then a downhill process. I5 represents a local minimum on the T_1_ surface, in which a hydrogen radical interacts non‐covalently with the second water molecule. Following the two highest lying alpha‐spin molecular orbitals in Figure 6b along the reaction coordinate illustrates the formation of the H radical, with the 3 s electron acting largely as a spectator. The excited 3p electron gradually moves away from the metal and localizes in an antibonding σ* orbital along an O−H bond, which is weakened. Once the H atom has evolved, around halfway through the rearrangement (0.5 of the interpolation coordinate), the unpaired electrons do not interact much, and the energies of T_1_ and S_0_ converge. From I5, the H atom may either evaporate in T_1_, or a second ISC to S_0_ takes place. In singlet spin multiplicity, the H radical can either attack the second water molecule and form H_2_, or recombine with the hydroxide moiety and eliminate H_2_O.

In this photochemical scenario, the [HAlOH(H_2_O)]^+^ structure, which is pivotal for thermally activated H_2_ elimination in larger cluster, does not play a role. The situation may change for larger clusters, for which such detailed excited state calculations were not performed. However, we think it is not too speculative to argue that H atom elimination involves ISC and gradual transfer of the excited 3p electron to an antibonding orbital of a water ligand to weaken and finally break the O−H bond.

## 5 Vanadium

### 5.1 Ground‐state reactivity

As a first‐row transition metal, vanadium has several stable oxidation states, which leads to quite interesting chemistry when [V(H_2_O)_*n*_]^+^ ions are exposed to room temperature black‐body radiation.^[82]^ In this case, H atom elimination is observed for *n*=9–12, and it is the dominant dissociation pathway for *n*=9–11. Molecular hydrogen H_2_ is formed over a wider range, *n*=9–23, *n*≠15. It is tempting to transfer the mechanisms of H and H_2_ formation from magnesium and aluminum, respectively. UV/Vis spectra of [V(H_2_O)_*n*_]^+^, however, show maximum absorption above 2 eV, significantly above the range observed for hydrated electron,^[36]^ and calculated structures containing a hydrated electron lie about 20 kJ mol^−1^ higher in energy for *n*≥9. The UV/Vis spectra also show unambiguously that, in contrast to [Al(H_2_O)_*n*_]^+^, the non‐inserted [V(H_2_O)_*n*_]^+^ species have a substantial lifetime for *n*≤12, and are present in the ICR cell after trapping is completed.^[36]^ Thermochemical analysis of the H atom elimination reaction^[83]^ shows that it is slightly lower in energy than H_2_O evaporation for *n*=9, the onset of H elimination, for the intact [V(H_2_O)_*n*_]^+^ species. In contrast, H atom loss from inserted [HVOH(H_2_O)_*n*‐1_]^+^ is too energetically demanding, so that the competing water loss prevails in a thermally activated reaction.^[83]^ The precursor for H atom elimination is definitely the non‐inserted [V(H_2_O)_*n*_]^+^ species.

**Figure 7:**
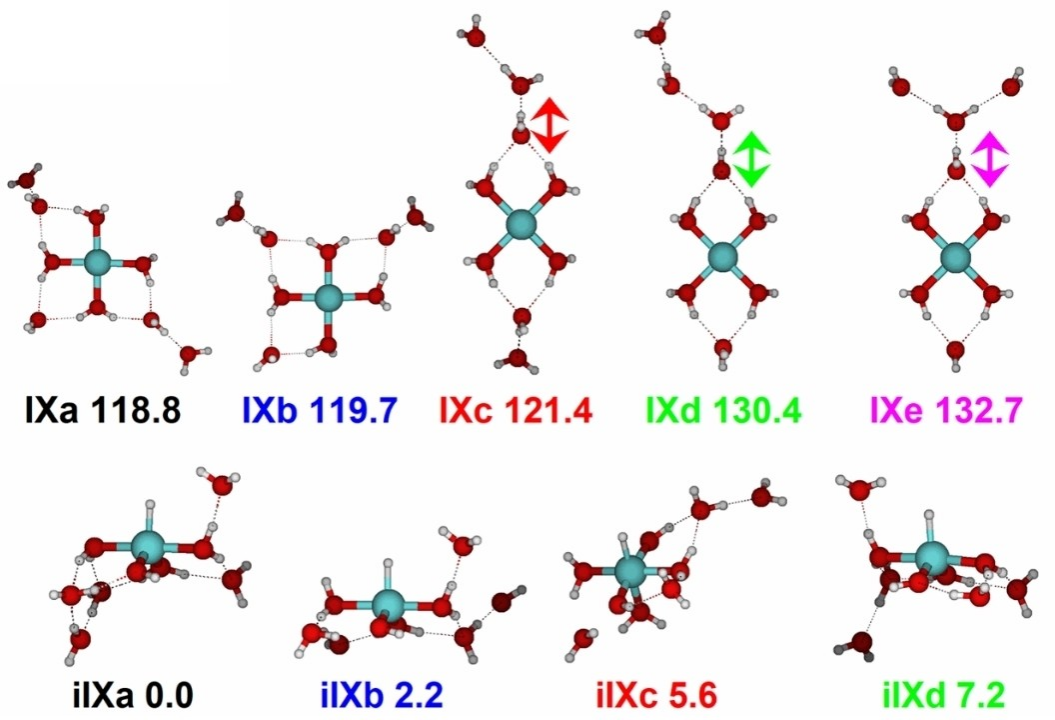
Intact and inserted isomers of [V(H_2_O)_9_]^+^ along with relative energies in kJ mol^−1^ (B3LYP/aug‐cc‐pVDZ). Arrows indicate the most red‐shifted O−H stretch, in each case a water molecule in a double acceptor binding motif in the second solvation shell. Reproduced under CC‐BY 3.0 license from Ref. [83]. © 2022 The Authors.

Figure 7 shows calculated structures of intact and inserted isomers for *n*=9. For the intact species, the V^+^ ion features a square‐planar coordination, consistent with a high‐spin complex of a metal center with 3d^4^ configuration.^[84]^ The disk‐like arrangement of water molecules in higher solvation shells, however, is very delicate, since the hydrogen bonds to the second‐shell water molecules are quite strained. Isomers with long water chains are energetically competitive, and are populated under the conditions of the experiment. These water chains afford attack of the metal center by an outer‐shell water molecule, which leads to two conceivable scenarios:

1. If a hydrogen atom attacks the metal center, a concerted proton transfer similar to the first stage of the aluminum ground‐state reaction can lead to formation of the HVOH^+^ hydride‐hydroxide core, which is highly exothermic. Instead of staying as a hydride ligand, however, the H atom may evaporate spontaneously by taking away the excess energy released during the hydride formation. This in line with BIRD or IRMPD experiments that no multiple water loss was observed.[^82^, ^83^] There are two important differences to the aluminum case; (i) H atom elimination is thermochemically accessible, and (ii) formation of H^−^ involves spin change of an electron, since all four valence electrons in the high‐spin complex are unpaired.
2. The alternative scenario is an oxygen atom coordinating to the metal center, which destabilizes the electron in the 3d_z_^2^ orbital. Similar to the situation in [Mg(H_2_O)_*n*_]^+^, *n*≥6, the electron gets displaced from the metal center, but the number of water molecules is not yet sufficient to stabilize a solvated electron. Instead, the electron localizes in an antibonding O−H orbital, which destabilizes the bond and triggers H atom elimination. One may speculate that in this case, H_2_ elimination may proceed by radical abstraction from a second first‐shell water ligand, following a similar pathway as in the photochemistry of Al^+^(H_2_O)_2_.

For clusters with *n* >12, however, the insertion reaction seems to proceed analogously to ground‐state hydrated aluminum, since no intact [V(H_2_O)_*n*_]^+^ are observed in the UV/Vis experiments.^[36]^ We think that the clusters form by pickup of V^+^ in a pre‐formed water cluster. In such large clusters, it may become impossible to form the square‐planar configuration of V^+^ in the quintet state. Five or six coordination structures could be formed and provide a more flexible water network for bringing hydrogen atoms toward the vanadium center. Concerted proton transfer upon uptake of V^+^ in the cluster may result in the inserted [HVOH(H_2_O)_*n*‐1_]^+^ species. Formation of molecular hydrogen then proceeds with the mechanism described above for [HAlOH(H_2_O)_*n*‐1_]^+^.

### 5.2 Photochemistry

The photodissociation spectra of [V(H_2_O)_*n*_]^+^ are broad.^[36]^ Weak d‐d transitions can be assigned for *n*=1 starting at 1.9 eV and redshift below 1 eV for *n*=3. The strong 3d–4p transitions^[85]^ start around 3.7 eV, and gradually shift to 2 eV for *n*=4. With completion of the square‐planar coordination of the V^+^ center,^[86]^ the redshift stops. Formation of atomic and molecular hydrogen is observed starting with *n*=1. The inserted [HVOH(H_2_O)_*n*‐1_]^+^ species do not absorb in the studied wavelength range.

**Figure 8:**
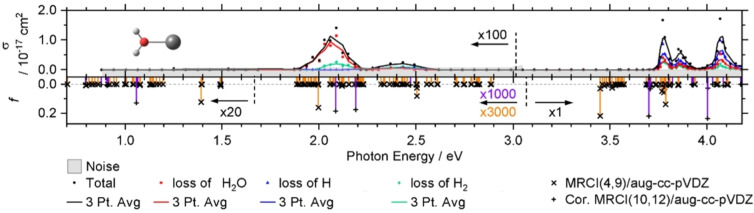
Experimental photodissociation cross section for V^+^(H_2_O) decomposed into different reaction channels. Weak transitions are scaled as indicated for better readability. The corresponding calculated oscillator strength *f* is shown as vertical lines, including spin‐orbit coupling on the MRCI/aug‐cc‐pVDZ//B3LYP/aug‐cc‐pVDZ level of theory. The MRCI(10,12) calculation of quintet states includes the Davidson correction. Reproduced under CC‐BY 3.0 license from Ref. [36]. © 2021 The Authors.

A closer look at the spectrum for *n*=1, Figure 8, reveals a quite distinct energy dependence of the photodissociation channels. At the lowest energies, only H_2_O loss is observed, and this channel is dominant in the first weak 3d/4s–3d/4 s band, although H_2_ evolution kicks in at 2 eV. In the second, weaker 3d/4s–3d/4 s band, however, H_2_ formation is already slightly more pronounced than water loss. In the strong 3d/4s–4p band starting at 3.7 eV, H atom formation is suddenly the prevailing channel, while water loss and H_2_ evolution occur in roughly equal abundance. With increasing energy, however, water loss becomes more important, and at 4.1 eV photon energy, the order of partial cross sections for the three elimination reactions is *σ*(H)>*σ*(H_2_O)>*σ*(H_2_). Comparison with CID results from the Armentrout group again confirms that H and H_2_ evolution are genuinely photochemical reactions.^[20]^

**Figure 9:**
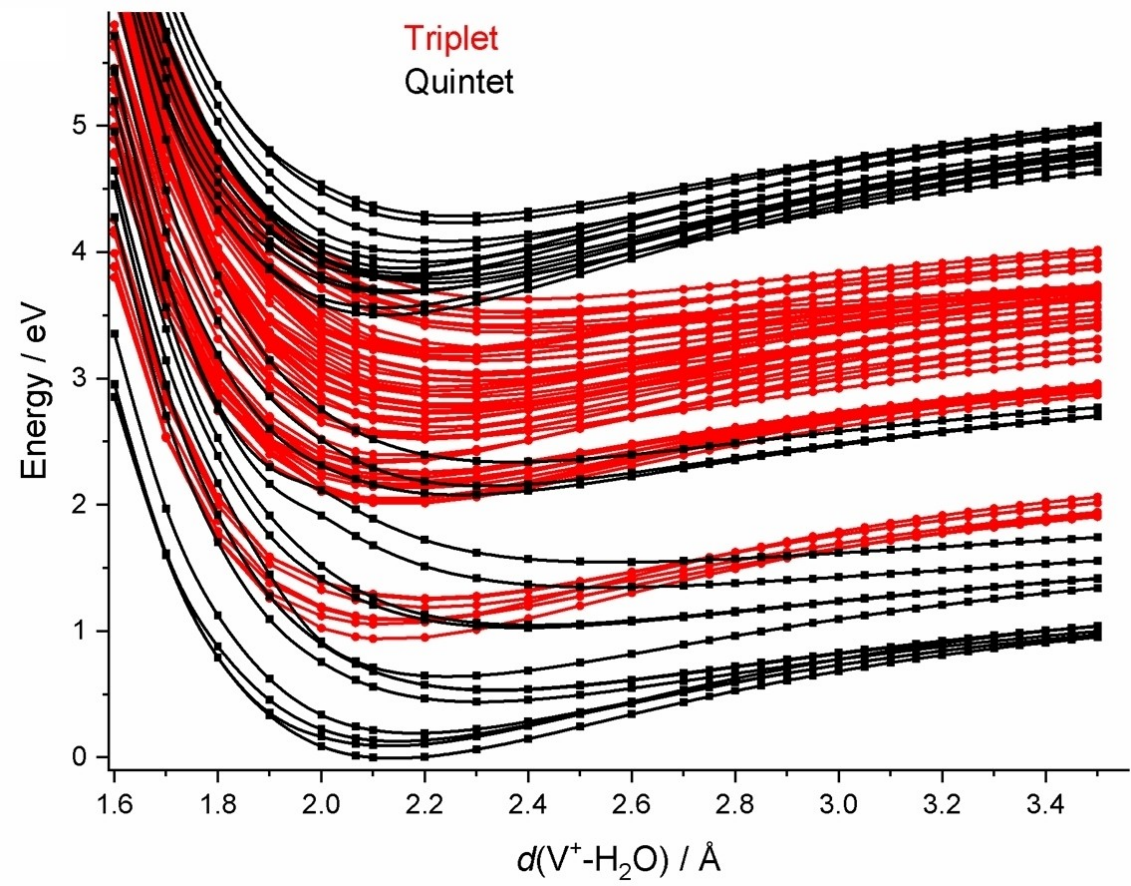
Splined potential energy surface scans for the relevant quintet and triplet states along the water dissociation coordinate *d*(V^+^−(H_2_O)) on the CASSCF(4,9)/aug‐cc‐pVDZ level of theory. All other internal coordinates are kept at the values calculated for the V^+^(H_2_O) minimum. Reproduced under CC‐BY 3.0 license from Ref. [36]. © 2021 The Authors.

Identification of the relevant excited states that contribute to the observed photochemistry is made difficult by the sheer number of excited states in this transition metal complex. Figure 9 shows potential energy curves along the V−O dissociation coordinate of quintet and triplet states that are accessible with the experimental photon energies. With increasing energy, a sparse manifold of quintet states is intersected by five triplet state up to around 2 eV. Above 2 eV, a dense manifold of triplet states emerges, which features some conical intersections to a relatively small number of quintet states. Only above 3.5 eV, another manifold of quintet states sets in, which overlaps within about 0.5 eV with the triplet state manifold. One has to keep in mind that this is only one of six internal coordinates of the complex, and additional conical intersections may be accessible for different coordinates. Nevertheless, this plot gives an idea about the complexity of the problem, and at the same time allows for a qualitative interpretation of the observed photochemistry.

**Figure 10:**
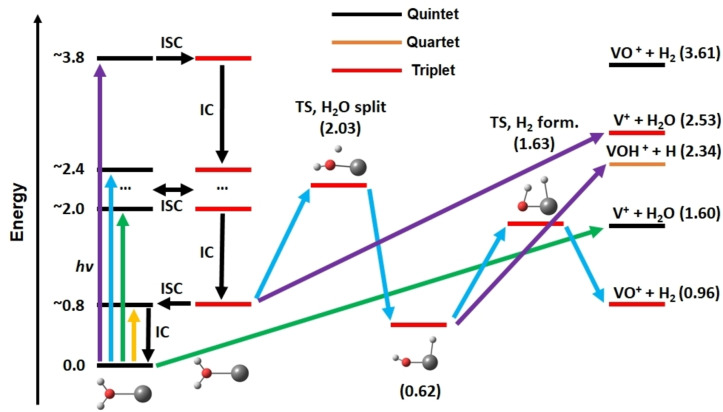
Schematic photochemical pathways in the V^+^(H_2_O) system involving internal conversion (IC) and intersystem crossing (ISC) and decomposition in low‐lying quintet or triplet states; relative energies are given in eV as calculated at MRCI(10,12)/aug‐cc‐pVDZ (excitation energies) and B3LYP/aug‐cc‐pVDZ (isomers, asymptotes) levels. Four different ranges for excitation are considered: For lowest energies (<1.5 eV, yellow), no fragmentation is possible; increased excitation energies (green, ∼2.0 eV) allow for water loss, water splitting becomes accessible with more energy (blue, ∼2.4 eV) in the triplet manifold through two transitions states (TSs). At high energies (purple, ∼3.8 eV), hydrogen radical loss and water elimination become increasingly important; note that H dissociation might also proceed from the quintet state (not shown for simplicity). Reproduced under CC‐BY 3.0 license from Ref. [36]. © 2021 The Authors.

The system can relax via internal conversion and intersystem crossings through conical intersections from any excited state to low‐lying triplet and quintet states, most likely even to the lowest‐lying states. Expanding on the work of Ugalde and co‐workers,^[87]^ we calculated transition states for V insertion into the O−H bond and H_2_ elimination, as well as asymptotes for H atom elimination, in the quintet ground state and lowest‐lying triplet state.^[36]^ Figure 10 summarizes the results qualitatively and illustrates the accessible pathways at different photon energies. At the photon energies below 1.5 eV, where two relatively strong transitions are predicted by the calculations in Figure 8, the available energy is not sufficient for any of the dissociation pathways, and the system relaxes most likely via infrared emission following internal conversion to the ground state, but fluorescence cannot be ruled out. The lowest accessible photodissociation channel is water evaporation from the quintet electronic ground state, which can be reached through a cascade of IC and ISC events. The minimum energy pathway to H_2_ evolution proceeds through two transition states on the lowest‐lying triplet PES. In the first transition state, we observe H atom transfer to the metal center or, in other words, insertion of the metal atom into the O−H bond, while the second one corresponds to formation of the H−H bond. Observation of H_2_ elimination at slightly higher photon energies than H_2_O loss is direct experimental evidence for ISC to the triplet manifold and radiationless relaxation to the lowest‐lying triplet state.

Two scenarios, which may both contribute, can explain the dominant H atom loss in the 3d/4s–4p band: after arriving in the lowest‐lying triplet PES, the barrierless H atom loss from ^3^V^+^(H_2_O) competes with the tight TS1 for water splitting, which lies slightly lower in energy. Entropy may favor H atom elimination. The same argument applies after formation of the HVOH^+^ structure on the triplet PES, where again H atom loss via a loose transition state is entropically favored over H_2_ elimination through a tight transition state; H atom loss in a quintet state seems less plausible, because in this case water loss is both barrierless and energetically preferred. Interestingly, a similar argument explains the renewed prevalence of H_2_O loss over H_2_ elimination. V^+^(H_2_O) in the triplet state may directly dissociate into V^+^ and H_2_O before going through the tight transition state for splitting of H_2_O, which adds to the statistical weight of this pathway. On the other hand, H_2_ elimination on the quintet surface remains energetically out of reach.

Due to the many electronic states involved, this discussion on the basis of the lowest‐lying triplet and quintet surfaces can rationalize the observations, but different pathways in higher‐lying electronic states cannot be ruled out. It is also difficult to extrapolate these findings to clusters *n*>1, in particular with respect to H_2_ formation, where the presence of a second water molecule offers additional pathways. We therefore calculated possible reaction pathways on the lowest‐lying triplet surface for *n*=2‐4.^[36]^ In each case, the rate‐limiting step is insertion of the metal into an O−H bond. For the resulting [HVOH(H_2_O)_*n*‐1_]^+^ structure, energetically low‐lying transition states are available through which the hydride attacks a second water ligand to form H_2_ and hydrated vanadium dihydroxide.

## 6 Discussion

At first sight, one might expect that hydrogen evolution at metal centers follows a uniform mechanism. The detailed analysis above illustrates that the opposite is the case. In Table 1, we attempt to summarize and categorize the identified mechanisms, but this survey is surely not complete. Open questions are the photochemical mechanisms for hydrogen evolution in larger clusters, in particular for vanadium.

**Table 1:** Overview of reactivity in [M(H_2_O)_*n*_]^+^ clusters, M=Mg, Al, V, in the electronic ground state and in electronically excited states.

Metal	Ground State	Excited State
Mg	H loss, *n*=6–21: recombination of H^+^ with hydrated electron	H loss, *n*=1–5: transfer of 3p electron to antibonding σ* orbital along O−H bond
Al	H_2_ loss, *n*=11–24: two‐step mechanism; formation of HAlOH^+^ via concerted proton transfer, then recombination of H^+^ with H^−^ via a second concerted proton transfer	H loss, *n*=1–4, 6, 7 H_2_ loss, *n*=2–8 *n*=2: ISC from singlet to triplet, transfer of 3p electron to antibonding σ* orbital along O−H bond, H radical is eliminated or attacks a second water molecule to yield H_2_, no insertion
V	H loss, *n*=9–12: H evaporation upon formation of [HVOH(H_2_O)_*n*‐1_]^+^ structure via concerted proton transfer or direct O−H bond cleavage induced by transfer of 3d_z_^2^ electron (tentative mechanism) H_2_ loss, *n*=9–12: radical abstraction after formation of H atom in [V(H_2_O)_*n*_]^+^ structure (tentative mechanism) *n*=9–23, *n*≠15: formation of HVOH^+^ in ion source, recombination H^+^ with H^−^ via concerted proton transfer	H loss, *n*=1–12 H_2_ loss, *n*=1‐3 (strong), *n*=4–12 (weak) *n*=1: ISC from quintet to triplet, followed by formation of HVOH^+^, H, H_2_ elimination on triplet surface *n*>1: ISC is plausible since density of states is higher in the triplet manifold; H_2_ energetically possible only in triplet state, H elimination possible in quintet ground state; many pathways energetically accessible

Atomic hydrogen may form in the electronic ground state by recombination of a proton with a hydrated electron, displaced from the metal center, as observed for magnesium. With vanadium, however, the metal center is involved as electron donor, and most likely the H atom is transferred to the metal before it can leave. Alternatively, the 3d_z_^2^ electron may weaken the O−H bond upon transferring into its antibonding σ* orbital. Ground state molecular hydrogen formation is following the same mechanism for aluminum and at least the larger vanadium species, with hydride‐metal‐hydroxide formation followed by hydride‐proton recombination.

Excited state chemistry, however, affords direct H atom expulsion from a first shell water molecule, definitely without involving H atom transfer to the metal center for Mg^+^(H_2_O) and Al^+^(H_2_O)_2_. Electron transfer from the metal center to an antibonding O−H molecular orbital weakens the O−H bond and enables H atom elimination. This mechanism was established by detailed analysis of the excited state reactions path for Al^+^(H_2_O)_2_, and it is probably also valid for Mg^+^(H_2_O)_*n*_, *n*=1–5. For vanadium, on the other hand, H atom transfer to the metal most likely precedes H atom elimination. Photochemistry of Al^+^(H_2_O)_2_ and V^+^(H_2_O) involves ISC, but the high density of states in the case of the transition metal vanadium may open a larger variety of reaction paths on several excited state potential energy surfaces.

Photochemical H_2_ elimination does not require the extensive hydrogen bonded network as the ground state two‐step mechanism. The detailed mechanism, however, is specific for the metal: aluminum is oxidized to the dihydroxide ion Al(OH)_2_^+^ by radical abstraction. With vanadium, the metal‐bound hydride formed after ISC attacks another first‐shell water molecule while still bound to the metal center.

## 7 Conclusions and Outlook

We have identified five different mechanisms of hydrogen evolution at metal centers, both in the electronic ground state as well as excited state photochemistry. Hydrogen evolution clearly has many interesting facets and comes in subtle shades, thus any attempt to generalize our findings based on these three metals seems premature, and some mechanistic details are still subject to critical evaluation. Metal insertion into the O−H bond does take place, and the formed hydride facilitates H_2_ evolution in the presence of a larger number of water molecules by a concerted proton transfer. However, this is not a necessary condition, direct H elimination from first‐shell water ligands or proton recombination with a hydrated electron proceed without the insertion step, and the hydrogen radical may abstract a hydrogen atom from a second water molecule to form H_2_.

To obtain a more complete picture of these prototypical hydrogen evolution reactions, the work needs to be extended to a wider variety of metals, and also to water clusters containing more than one metal center. This will be particularly challenging for theory: such systems may quickly become too complex for a thorough ab initio‐based treatment, especially when transition metals are involved.

## Conflict of interest

The authors declare no conflict of interest.

8

## Biographical Information

*Milan Ončák is a theoretical chemist working as an assistant professor at the University of Innsbruck. He obtained his Ph.D. with Petr Slavíček (University of Chemistry and Technology, Prague), followed by an Alexander von Humboldt Fellowship with Joachim Sauer (Humboldt University Berlin) and a Lise Meitner Fellowship with Martin Beyer (University of Innsbruck). His research interests include spectroscopy of transition metals, (photo)chemistry of organic molecules in water/salt environment as models of aerosol particles, influence of solvent on photochemistry, and statistical modeling of photoinduced processes*.

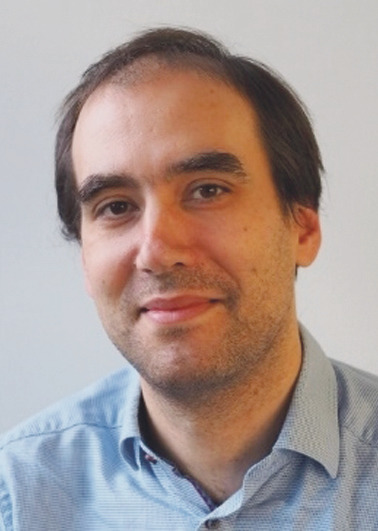

## Biographical Information

*Chi‐Kit (Andy) Siu obtained his Ph.D. with Zhifeng Liu at the Chinese University of Hong Kong. He was an Alexander von Humboldt Postdoctoral Fellow with Vladimir Bondybey at TU Munich. After a postdoctoral stay with K. W. Michael Siu and Alan C. Hopkinson (York University, Canada), he joined City University of Hong Kong and is currently an Associate Professor in the Department of Chemistry. His research focuses on understanding mechanisms, thermodynamics and kinetics of unimolecular and ion‐molecule reactions of gas‐phase peptide ions and hydrated clusters. Siu is currently serving as the President of the Hong Kong Society of Mass Spectrometry*.

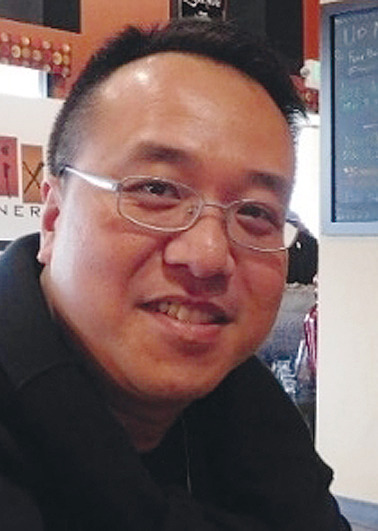

## Biographical Information

*Christian van der Linde received his Diploma in Chemistry in 2009 and his PhD in Physical Chemistry in 2012 from Kiel University, Germany. After graduation, he worked 2013–2014 as a postdoc in the group of James M. Lisy at the University of Illinois at Urbana‐Champaign, USA, on IRMPD of small alkali‐metal based clusters. Since 2015, he works as postdoc, and since 2019 as senior scientist at the University of Innsbruck, Austria, in the group of Martin Beyer, combining mass spectrometry and spectroscopy methods to study ion chemistry, spectroscopy and photochemistry various cluster systems*.

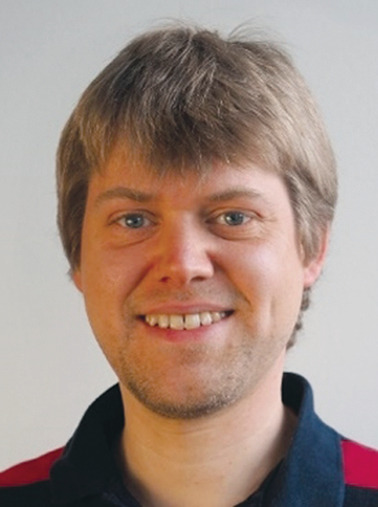

## Biographical Information

*Wai Kit Tang received his BS degree from Universiti Sains Malaysia (2008). After spending several years in industry, he obtained his PhD degree in Computational Chemistry (2017) under the supervision of Chi‐Kit Siu, City University of Hong Kong. He continued to work as a Postdoctoral Research Associate in the same group for four years before joining Vivian Wing‐Wah Yam's research group to conduct DFT studies on excited states of OLED materials. Currently, he is serving as a Senior Lecturer at Universiti Malaya, Malaysia. His research aims to understand spectroscopic properties, reaction mechanisms and excited states of metal‐containing molecular clusters*.

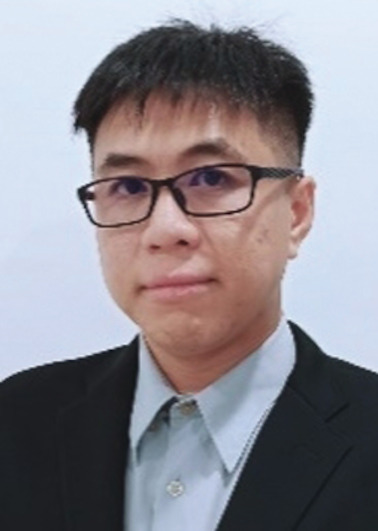

## Biographical Information

*Martin Beyer studied physics and obtained a PhD in physical chemistry at TU Munich. After staying at UC Berkeley with a Feodor Lynen postdoctoral fellowship (Alexander von Humboldt foundation), he received the Heinz Maier Leibnitz award (2003, DFG/BMBF) and obtained his venia legendi from TU Munich (2004). He moved with a DFG Heisenberg Fellowship to TU Berlin (2005) and held a professor position at Kiel University (2007‐2013) before moving to the University of Innsbruck. His research focuses on chemistry, spectroscopy and photochemistry of molecular ions and ionic clusters, chemical ionization methods for gas analysis, and fundamental concepts in mechanochemistry*.

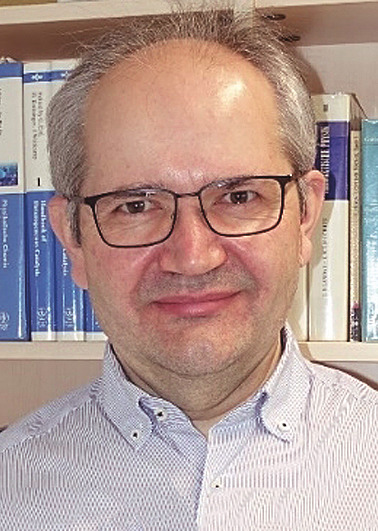

## Data Availability

Data sharing is not applicable to this article as no new data were created or analyzed in this study.
